# Development of Monoclonal Antibody and Diagnostic Test for Middle East Respiratory Syndrome Coronavirus Using Cell-Free Synthesized Nucleocapsid Antigen

**DOI:** 10.3389/fmicb.2016.00509

**Published:** 2016-04-20

**Authors:** Yutaro Yamaoka, Shutoku Matsuyama, Shuetsu Fukushi, Satoko Matsunaga, Yuki Matsushima, Hiroyuki Kuroyama, Hirokazu Kimura, Makoto Takeda, Tomoyuki Chimuro, Akihide Ryo

**Affiliations:** ^1^Department of Microbiology, School of Medicine, Yokohama City UniversityYokohama, Japan; ^2^Isehara Research Laboratory, Technology and Development Division, Kanto Chemical Co., Inc.Isehara, Japan; ^3^Department of Virology III, National Institute of Infectious DiseasesMusashimurayama, Japan; ^4^Department of Virology I, National Institute of Infectious DiseasesMusashimurayama, Japan; ^5^Division of Virology, Kawasaki City Institute for Public HealthKawasaki, Japan; ^6^Infectious Disease Surveillance Center, National Institute of Infectious DiseasesMusashimurayama, Japan

**Keywords:** MERS-coronavirus, nucleocapsid, antigen, detection, diagnosis, monoclonal antibody, cell-free protein synthesis

## Abstract

Protein nativity is one of the most critical factors for the quality of antigens used as immunogens and the reactivities of the resultant antibodies. The preparation and purification of native viral antigens in conventional cell-based protein expression systems are often accompanied by technical hardships. These challenges are attributable mainly to protein aggregation and insolubility during expression and purification, as well as to very low expression levels associated with the toxicity of some viral proteins. Here, we describe a novel approach for the production of monoclonal antibodies (mAbs) against nucleocapsid protein (NP) of the Middle East respiratory syndrome coronavirus (MERS-CoV). Using a wheat germ cell-free protein synthesis system, we successfully prepared large amounts of MERS-CoV NP antigen in a state that was highly soluble and intact for immunization. Following mouse immunization and hybridoma generation, we selected seven hybridoma clones that produced mAbs with exclusive reactivity against MERS-CoV NP. Epitope mapping and subsequent bioinformatic analysis revealed that these mAbs recognized epitopes located within relatively highly conserved regions of the MERS-CoV amino-acid sequence. Consistently, the mAbs exhibited no obvious cross-reactivity with NPs derived from other related viruses, including SARS coronavirus. After determining the optimal combinations of these mAbs, we developed an enzyme-linked immunosorbent assay and a rapid immunochromatographic antigen detection test that can be reliably used for laboratory diagnosis of MERS-CoV. Thus, this study provides strong evidence that the wheat germ cell-free system is useful for the production of diagnostic mAbs against emerging pathogens.

## Introduction

Middle East respiratory syndrome coronavirus (MERS-CoV), a novel human coronavirus, was first isolated in 2012 in the Arabian Peninsula (Zaki et al., 2012). MERS-CoV is a positive-sense, enveloped, single-stranded RNA virus of genus *Betacoronavirus* within subfamily *Coronavirinae* (de Groot et al., 2013). MERS-CoV infection often causes fever, cough, and severe pneumonia, occasionally accompanied by renal disease (Banik et al., 2015). More than 1600 laboratory-confirmed cases with high fatality rates (∼36% mortality) have been reported (World Health Organization [WHO], 2016). Because there is currently no specific antiviral drug or vaccine approved for clinical use against MERS-CoV, rapid diagnostic tests are urgently required to manage and control this virus. Indeed, rapid and specific diagnosis is essential for preventing the spread of any kind of infectious disease.

At present, laboratory testing for MERS-CoV is performed by quantitative reverse transcription-PCR assay (qRT-PCR) and RT-loop-mediated isothermal amplification (RT-LAMP) (Corman et al., 2012a,b; Shirato et al., 2014). These tests can detect nucleic acids derived from MERS-CoV in clinical respiratory, serum, and stool specimens. These nucleic acid-based tests require molecular techniques and specialized equipment, and are thus not suitable for point-of-care testing (POCT) or bedside diagnosis. Therefore, it is necessary to develop alternative methods that can be adapted to rapid and reliable clinical detection of MERS-CoV antigen, including enzyme-linked immunosorbent assay (ELISA) and immunochromatographic test (ICT).

Middle East respiratory syndrome coronavirus comprises four structural proteins: spike (S), envelope (E), membrane (M), and nucleocapsid (N) (van Boheemen et al., 2012). S protein is a major component of the viral surface that binds dipeptidyl peptidase 4 (DPP4), enabling the virus to enter and infect cells (Raj et al., 2013). Therefore, S protein is considered to be a prospective therapeutic and diagnostic target (Song et al., 2013; Jiang et al., 2014; Zhang et al., 2014; Li et al., 2015). However, because neutralizing antibodies mainly target this antigen, coronaviruses express several mutant forms of S protein in order to escape the immune response and achieve viral persistence (Tang et al., 2014). On the other hand, amino-acid mutations in N protein are much less common (Wernery et al., 2015). N protein is produced at high levels within infected cells, and is thus a promising candidate target for clinical diagnosis (Lau et al., 2004; He et al., 2005; Kogaki et al., 2005; Liang et al., 2013; Chen et al., 2015).

N protein functions in packaging the viral genomic RNA to form the helical nucleocapsid, as well as in viral transcription and assembly (McBride et al., 2014). It has three distinct and conserved domains: the N-terminal domain (NTD), linker region (LKR), and C-terminal domain (CTD) (McBride et al., 2014). The NTD of human coronavirus N protein contains highly conserved motifs (Yu et al., 2005; Chang et al., 2014). To prevent cross-reactivity with other human coronaviruses and specifically detect MERS-CoV, it is necessary to develop antibodies that target non-conserved regions. However, the viral structural protein is generally unstable and insoluble in its monomeric or oligomeric forms, making it difficult to prepare antigen for immunization. Moreover, refolding of solubilized viral proteins by denaturing agents often results in misfolding and functional loss (Schein, 1991). To overcome these problems, we recently developed a cell-free based viral protein production system using wheat germ extract (Matsunaga et al., 2014). Because wheat is a eukaryote, this system can synthesize properly folded and biologically active viral proteins equivalent to those expressed in mammalian cells (Endo and Sawasaki, 2005, 2006; Goshima et al., 2008).

In this study, we synthesized recombinant MERS-CoV N protein (MERS-NP) and raised monoclonal antibodies (mAbs) that could specifically detect this protein. We also describe the development and evaluation of a rapid test format including ELISA and ICT that can be used in POCT for MERS-CoV infection.

## Materials and Methods

### Expression Plasmid

Complementary DNAs encoding nucleocapsid proteins (NPs) of human coronaviruses (MERS-CoV, GenBank No. NC_019843; SARS-CoV, GenBank No. NC_004718; HCoV-HKU1, GenBank No. NC_006577; HCoV-OC43, GenBank No. NC_005147; HCoV-229E, GenBank No. NC_002645; HCoV-NL63, GenBank No. NC_005831) were synthesized by GENEWIZ (South Plainfield, NJ, USA). Synthetic cDNAs were digested with *Xho*I and *Kpn*I and inserted into pEU-E01-His-TEV-MCS and pcDNA3-HA-MCS. To generate the expression vector for antigen production in the wheat germ cell-free system, the MERS-NP open reading frame encoding amino acids 122–413 was amplified by PCR using the forward primer 5′-GAGAGATATCTGGGTGCATGAGGACGGAG-3′ and the reverse primer 5′-GAGAGATATCTCAGTCTGTGTTCACATCG-3′. The amplified fragment was cloned into the *Eco*RV site of vector pEU-E01-His-TEV-MCS (CellFree Sciences, Yokohama, Japan). Deletion mutants of MERS-NP for epitope mapping were generated using the PrimeSTAR Mutagenesis Basal kit (Takara Bio, Otsu, Japan). The R395H mutation was introduced into MERS-NP using the PrimeSTAR Mutagenesis Basal kit (Takara Bio, Otsu, Japan).

### Cell-free Protein Synthesis and Purification

*In vitro* transcription and cell-free protein synthesis were performed as previously described (Takai and Endo, 2010; Takai et al., 2010; Senchi et al., 2013; Matsunaga et al., 2014, 2015). For cell-free protein synthesis, WEPRO7240H wheat germ extract (CellFree Sciences, Yokohama, Japan) was used in the bilayer translation method as previously described (Matsunaga et al., 2014). Synthesized proteins were confirmed by immunoblotting.

His-MERS-NP (122-413) protein, used for mouse immunization, was synthesized using a Protemist XE robotic protein synthesizer (CellFree Sciences, Yokohama, Japan). The cell-free translation reaction mixture (6 ml) was separated into soluble and insoluble fractions by centrifugation at 15,000 rpm for 15 min. The soluble fraction was mixed with Ni-Sepharose High Performance beads (GE Healthcare, Waukesha, WI, USA) in the presence of 20 mM imidazole. The beads were washed three times with washing buffer [20 mM Tris-HCl (pH 7.5), 500 mM NaCl] containing 40 mM imidazole. His-MERS-NP (122–413) was then eluted in washing buffer containing 500 mM imidazole. Amicon Ultra centrifugal filters (Millipore, Bedford, MA, USA) were used to concentrate purified His-MERS-NP (122–413) by approximately 10–20-fold. Protein concentration was determined using the Bradford method, with bovine serum albumin (BSA) as a protein standard.

### Immunization and Generation of Hybridomas

Immunization of BALB/c mice and generation of hybridomas producing anti-MERS-NP antibody were carried out as previously described (Kimura et al., 1994, 1996; Matsunaga et al., 2014). Primary antibodies in hybridoma culture supernatant were tested by immunoblot analysis with non-tagged recombinant N protein. Animal experiments were performed ethically according to the Guidelines for Animal Experiments at Yokohama City University. All of the procedures were approved by the Committee on Experimental Animals at the Yokohama City University.

### Purification of mAbs

Hybridoma cells were grown in CD hybridoma medium AGT medium (Thermo Fisher Scientific, Rockford, IL, USA). Primary antibodies in the culture supernatant of each clone were separated by centrifugation at 8,000 rpm for 15 min and eluted with AcroSep Hyper DF columns (Pall, New York, NY, USA). Samples were then further concentrated 10–20-fold using Amicon Ultra centrifugal filters (Millipore, Bedford, MA, USA). Concentrations of purified IgG were determined by measuring the absorbance at OD_280_. Immunoglobulin characterization was carried out using the IsoStrip mouse monoclonal antibody isotyping kit (Roche Diagnostics, Basel, Switzerland).

### Homology Modeling of MERS-NP and Epitope Localization Analysis

The dimer model of MERS-NP was constructed by homology modeling based on the partial structure of SARS-NP (PDB code. 1SSK, Huang et al., 2004; PDB code. 2CJR, Chen et al., 2007) using the MODELLER9.15 software (Webb and Sali, 2014). Protein structures not registered in PDB were estimated by the I-TASSER and QUARK servers and used as templates for homology modeling (Xu and Zhang, 2012; Yang et al., 2015). Energy minimization of the generated model was carried out using Swiss PDB viewer4.1 (Guex and Peitsch, 1997). Surface localization of each epitope was determined using the UCSF Chimera software (Pettersen et al., 2004).

### Immunoprecipitation Analysis

Immunoprecipitation was performed as previously described (Miyakawa et al., 2015). Briefly, HEK293A cells were grown on a 100-mm dish for 24 h, and then transfected with HA-MERS-NP. Cell lysates were immunoprecipitated with EZview Red anti-HA Affinity Gel (Sigma–Aldrich) or 2 μg of each anti-MERS-NP antibody mixed with protein G-Sepharose (GE Healthcare, Little Chalfont, UK). Bound proteins were analyzed by immunoblotting.

### Selection of the Optimal Pair of mAbs for Sandwich ELISA

Each mAb was diluted in 50 mM of carbonate buffer (pH 9.6) to a concentration of 10 μg/mL, and then added to an ELISA plate (AGC TECHNO GLASS, Shizuoka, Japan). To immobilize the antibodies, the plate was incubated overnight at 4°C. Wells were blocked with PBS containing 2% (w/v) skim milk for 1 h at room temperature (RT). After three washes with PBS containing 0.05% (v/v) Tween-20 (PBS-T), 100 μL of antigen protein (1 ng/mL) diluted with PBS-T or blank (PBS-T alone) was added and incubated for 60 min at RT. After three washes with PBS-T, 100 μL of each mAb conjugated with horseradish peroxidase (HRP) was added into each well and incubated for 60 min at RT. Antibody labeling was performed using the Peroxidase Labeling Kit -NH_2_ (Dojindo Laboratories, Kumamoto, Japan). After three washes with PBS-T, 100 μL of ABTS substrate solution (Kirkegaard & Perry Laboratories, Washington, DC, USA) was added and incubated for 30 min at RT. Absorbance at 415/492 nm was measured on a plate reader, and the signal-to-noise ratio (S/N) was calculated.

### Selection of the Optimal Antibody Pair for ICT

For the test line, anti-MERS-NP antibodies #20, #29, and #46 were diluted in 50 mM of phosphate buffer (pH 8.0) to a concentration of 1 mg/mL and immobilized on a nitrocellulose membrane (Millipore, Bedford, MA, USA). To prepare the control line, an anti-mouse IgG antibody was diluted to 0.125 mg/mL and immobilized onto another area of the same membrane. The membrane was dried and blocked at RT, washed with deionized water, and lyophilized.

To produce conjugate pads, anti-MERS-NP mAbs #5, #13, and #20 were diluted in 50 mM of phosphate buffer (pH 8.0) to a concentration of 0.05 mg/mL and labeled with colloidal gold (Tanaka Kikinzoku Kogyo, Tokyo, Japan). The colloidal gold-conjugated mAbs were blocked with 0.5% casein (Kanto Chemical, Tokyo, Japan). After washing three times with phosphate buffer (pH 7.0), labeled mAbs were diluted to an OD_525_ 4.4 and impregnated into glass fibers (Millipore, Bedford, MA, USA). The glass fibers were lyophilized.

Immunochromatographic strips were generated by assembling a glass fiber (sample pad), conjugate pad, nitrocellulose membrane, and liquid absorbent pad. To compare each combination of antibodies, antigen proteins were diluted to 12.5 ng/0.1 mL in 40 mM phosphate buffer containing 150 mM NaCl and applied to the strips. The color intensity of red lines at the test and control position and the background of the membrane were visually observed and evaluated after a 15 min reaction.

### Bioinformatic Analysis

Homology of NPs among human coronaviruses (MERS-CoV, GenBank No. NC_019843; SARS-CoV, GenBank No. NC_004718; HCoV-HKU1, GenBank No. NC_006577; HCoV-OC43, GenBank No. NC_005147; HCoV-229E, GenBank No. NC_002645; HCoV-NL63, GenBank No. NC_005831) was analyzed by multiple sequence alignment using the MUSCLE software (Edgar, 2004).

To examine amino-acid variability among NPs of each MERS-CoV strain, 113 MERS-NP sequences from the GenBank were aligned using the MUSCLE software. A Shannon entropy score was calculated for each position in the protein alignment as previously described (Yang, 2009). Phylogenetic trees were generated via the maximum-likelihood method with 1000 bootstrap replicates using MEGA5 after removal of 100% identical sequences (Tamura et al., 2011). The dataset was analyzed using the Jones–Taylor–Thornton (JTT) amino-acid substitution model.

### Preparation and Quantitation of MERS-CoV

Prototype strain of MERS-CoV were provided by Drs. Ron A. M. Fouchier and Bart L. Haagmans (Erasmus Medical Center). MERS-CoV were propagated in Vero cells expressing TMPRSS2, as described previously (Shirato et al., 2013). Viral samples were concentrated by centrifugation from culture supernatant of MERS-CoV infected cells. MERS-CoV was inactivated by addition of Nonidet P-40 (NP-40) to a final concentration of 1% (v/v) prior to each immunoassay.

Viral RNA was extracted from the samples using the QIAamp viral RNA mini kit (Qiagen, Valencia, CA, USA). Quantitation of viral copy number was carried out by reverse-transcription droplet digital PCR (RT-ddPCR) using One-Step RT-ddPCR Advanced kit for Probes (Bio-Rad, Hercules, CA, USA). A 20 μL reaction was set up containing 2 μL of RNA (equivalent to 20 ng), 2 μL of a mixture of forward/reverse primers and probe, 1 μL of 300 mM DTT, 2 μL of reverse transcriptase, 8 μL of RNase-free water, and 5 μL of Supermix. Primers and probe sets for *Orf1a* were used as previously reported in qRT-PCR assays (Corman et al., 2012b); final concentrations of primers and probe were 900 and 250 nM, respectively. Droplets were formed in a QX200 droplet generator (Bio-Rad, Hercules, CA, USA). Thermal cycling conditions were as follows: 42°C for 60 min for the RT reaction; 95°C for 10 min; 40 cycles of 95°C for 30 s and 56°C for 1 min; and a final 10 min denaturation step at 95°C. After thermal cycling, plates were transferred to the QX200 droplet reader (Bio-Rad, Hercules, CA, USA). Positive droplets containing amplification products were discriminated from negative droplets by applying a fluorescence amplitude threshold in the QuantaSoft software (Bio-Rad, Hercules, CA, USA).

### Immunoblot Analysis

Cell-free synthesized proteins or cell culture supernatants containing inactivated MERS-CoV were mixed with an equal volume of 2X SDS sample buffer [125 mM Tris-HCl (pH 6.8), 4% SDS, 20% glycerol, 10% 2-mercaptoethanol and 0.01% bromophenol blue] and heated at 100°C for 5 min. After separation by 12.5% or 15% SDS-PAGE using Hi-QRAS Gel N (Kanto Chemical, Tokyo, Japan), the proteins were electrotransferred onto an Immobilon-P PVDF Transfer Membrane (Millipore, Bedford, MA, USA) as described previously (Nishi et al., 2014). The membrane was blocked in Tris-buffered saline (TBS) containing 2% (w/v) skim milk for 30 min, and then incubated for 1 h with anti-MERS-NP mAbs or anti-His polyclonal antibody (GTX115045; GeneTex, Irvine, CA, USA) in TBS containing 0.1% (v/v) Tween 20 (TBS-T; 1:1000 dilution) and 0.4% (w/v) skim milk. After three washes with TBS-T, the membrane was incubated for 60 min in PBS containing HRP-conjugated goat-anti mouse or rabbit IgG antibody (1:10000 dilution; GE Healthcare). After an additional three washes in TBS-T, proteins were detected with SuperSignal West Dura Extended Duration Substrate (Thermo Fisher Scientific, Rockford, IL, USA) or Immobilon (Millipore, Bedford, MA, USA) on a Lumi-Imager F1 (Roche Diagnostics, Basel, Switzerland).

### Detection Limit of Antigen-Capture ELISA

Sensitivity analysis of antigen-capture ELISA was carried out as described above with some modifications. Briefly, mAb (#46) was immobilized onto a plate at a concentration of 2.5 μg/mL. After the plate was blocked, inactivated MERS-CoV (1.2 × 10^6^ copies/0.1 mL) and recombinant antigen protein (1 ng/0.1 mL) were serially twofold diluted with PBS-T and subjected to analysis. After reaction with HRP-conjugated mAb (#20), a chromogenic reaction was conducted by adding 100 μL of TMB Substrate solution (Kirkegaard & Perry Laboratories, Washington, DC, USA) per well, followed by incubation for 5 min; the reaction was halted by the addition of 100 μL of 1 M H_2_SO_4_. Absorbance at 450/630 nm was measured on a plate reader.

### Detection Limit of ICT

Immunochromatographic detection of MERS-CoV was carried out as described above with some modifications in preparation of the conjugate pad. Conjugation of mAb #20 with colloidal gold was performed at a concentration of 0.2 mg/mL. After blocking and washing, conjugated pads impregnated with labeled antibody solution at an OD_525_ of 8.0.

Sensitivity of ICT was evaluated by adding serially twofold diluted inactivated MERS-CoV (1.2 × 10^7^ copies/0.1 mL) and recombinant antigen protein (2 ng/0.1 mL).

## Results

### Production of mAbs to Target Nucleocapsid Protein of MERS-CoV

**FIGURE 1:**
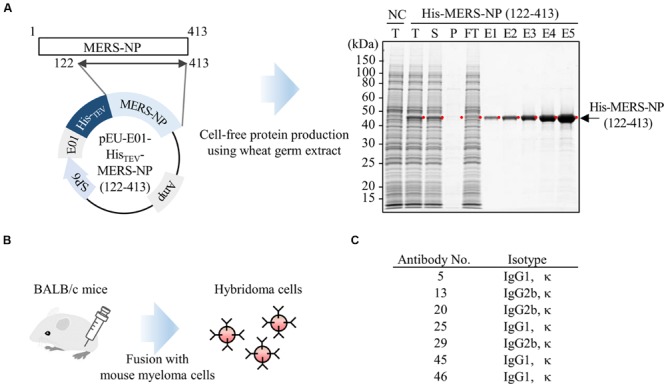
**Production of mAb using wheat germ cell-free synthesized nucleocapsid antigen. (A)** Schematic representation of antigen protein production. Recombinant Histidine (His)-tagged N-terminally truncated MERS-NP (122–413) was produced in a wheat germ cell-free system, and then purified using nickel-chelated Sepharose beads. Each protein fraction was analyzed by SDS-PAGE and visualized by CBB staining. Red dot and arrow indicate the target protein. NC, negative control; T, total fraction; S, supernatant; P, precipitate; FT, flow-through; E1–5, elution fractions 1–5. **(B)** Schematic diagram of hybridoma cells production generating anti-MERS-NP mAb. Purified His-MERS-NP (122–413) was injected into BALB/c mice. After 4 weeks, immunized mouse splenocytes were fused with myeloma cells, and 48 hybridoma cells were established. Of the 48 clones, seven exhibited relatively high reactivity to antigen proteins, as revealed by immunoblotting analysis, and were selected for further investigation. **(C)** List of selected hybridoma clones producing mAbs.

Previous studies described antigenic cross-reactivity among the NTDs of N proteins of human coronaviruses, including SARS-CoV (Yu et al., 2005). To minimize the cross-reactivity when generating mAbs, we produced N-terminally deleted MERS-NP (amino acids 122–413) as an antigen for antibody production. Complementary DNA encoding MERS-NP (122–413) was sub-cloned into pEU-His, a vector designed for expression of His-tagged proteins in the wheat germ cell-free system. As predicted, His-tagged MERS-NP (122–413) was expressed (**Figure 1A**). The protein was purified from the soluble fraction of the extracts using Ni-Sepharose beads followed by elution with imidazole.

The purified protein was used to immunize BALB/c mice. After 4 weeks, splenocytes were isolated and hybridomas were generated (**Figure 1B**). Ultimately, 48 stable hybridomas were obtained and designated #1–#48. Among the 48 clones, seven (#5, #13, #20, #25, #29, #45, and #46) were selected for further investigation based on their reactivity to MERS-NP in immunoblot analysis (**Figure 1C**).

### Epitope Analysis of Anti-MERS-NP mAb

**FIGURE 2:**
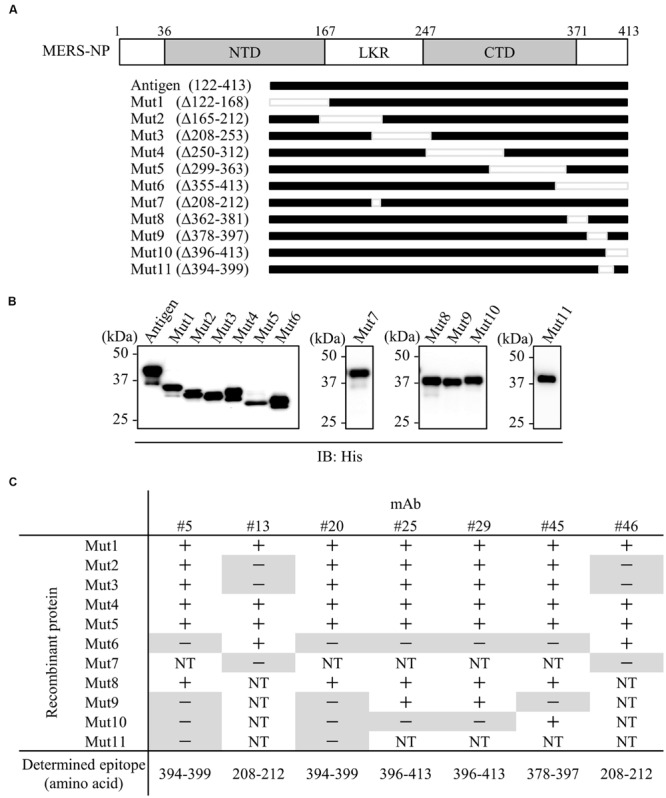
**Epitope mapping of mAbs. (A)** Schematic diagram of MERS-NP putative domain architecture and deletion mutants. For epitope mapping, 11 deletion mutants were produced as His-tagged proteins in the wheat germ cell-free system. NTD, N-terminal domain; LKR, flexible linker region; CTD, C-terminal domain. **(B)** Confirmation of protein expression. His-tagged NP and deletion mutants were immunoblotted with anti-His-tagged antibody. **(C)** Summary of epitope analysis. Reactivity of each mAb to deletion mutants was evaluated by immunoblotting. + and - indicate positive and negative detection, respectively. NT; not tested. Negative detections are highlighted in gray.

We next performed epitope mapping to determine the antibody binding sites. Using cell free-synthesized deletion mutants of MERS-NP, we carried out immunoblot analyses with the generated antibodies. In the first screen, we used six deletion mutants (Mut1-6; **Figures 2A,B**); the results revealed that two mAbs (#13 and #46) recognized the middle region corresponding to LKR, whereas the remaining five mAbs (#5, #20, #25, #29, and #45) bound the C-terminal end of the protein (**Figures 2A,B**). More precise epitope mapping was performed using five additional deletion mutants (Mut7-11; **Figures 2A,B**); the results are summarized in **Figure 2C**.

**FIGURE 3:**
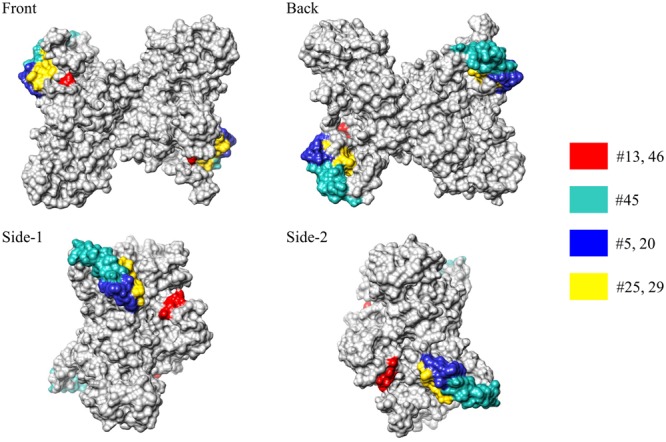
**Epitope localization in MERS-CoV-NP structural model.** Positions of epitopes in a structural model of dimer-forming MERS-CoV-NP, constructed by homology modeling using the structure of SARS-NP (PDB ID. 1SSK, 2CJR). Epitope localizations of each mAb are highlighted in different colors.

We next investigated whether the antigenic epitopes were located on the surface of MERS-NP. To this end, we used a previously reported solution structure of SARS-NP (PDB ID; 1SSK, 2CJR) for homology modeling of MERS-NP (Huang et al., 2004; Chen et al., 2007). Molecular modeling of MERS-NP using the UCSF Chimera software revealed that all mAb binding regions were located on the surface of N protein (**Figure 3**).

### Immunoprecipitation Assay

**FIGURE 4:**
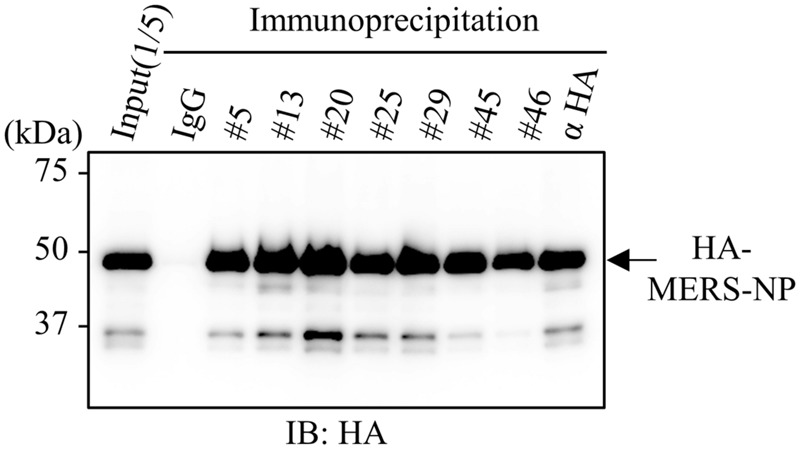
**Immunoprecipitation analysis with generated mAbs.** Immunoprecipitation analysis. HEK293A cells were transfected with plasmid vector encoding HA-tagged MERS-NP, and cell lysates were collected after 48 h. Samples were subjected to for immunoprecipitation with anti-MERS-NP antibodies followed by immunoblotting analysis with anti-HA antibody.

Next, because an antibody suitable for immunoprecipitation is likely to be conformation-sensitive, we examined whether our selected antibodies could be used in immunoprecipitation analysis (Mancia et al., 2007; Takeda et al., 2015). Cell lysates from HEK293A cells expressing HA-tagged MERS-NP were subjected to immunoprecipitation analysis with each selected antibody (**Figure 4**). The results revealed that all of the generated mAbs could be used for immunoprecipitation analysis.

### Screening for Appropriate Combinations of mAbs for Antigen-Capture ELISA and ICT

**FIGURE 5:**
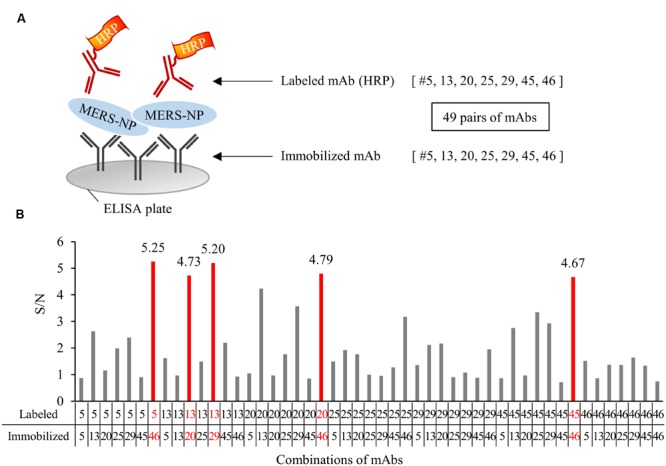
**Determination of optimal pairs of mAbs for antigen-capture ELISA. (A)** Schematic representation of sandwich ELISA. Each mAb was labeled with horseradish peroxidase (HRP) and subjected to ELISA analysis. 49 pairs of antibodies were tested. **(B)** Determination of the optimal combination of capturing and detection mAbs. S/N ratios for antigen detection by each of the 49 combinations were calculated in the presence of 0.1 ng/0.1 mL antigen V.S. blank. S/N values of selected five pairs were depicted on each bar.

**FIGURE 6:**
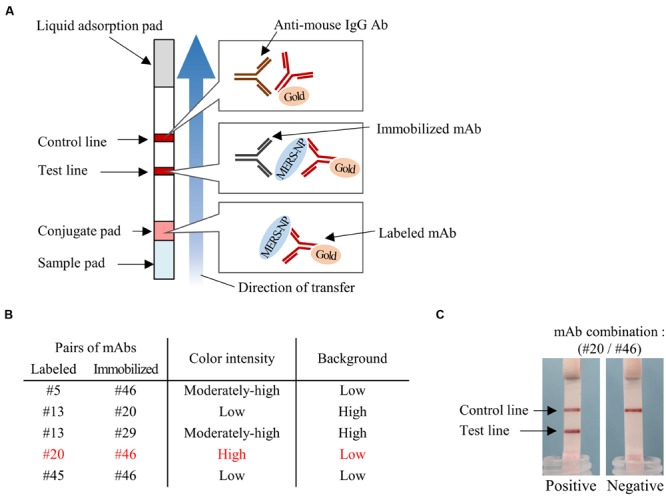
**Determination of optimal pairs of mAbs for ICT. (A)** Schematic diagram of rapid ICT. **(B)** Selection of appropriate pairs of mAbs for ICT. Color intensity of test line and background level were evaluated with antigen protein at 12.5 ng/0.1 mL. **(C)** Typical positive and negative results of ICT using the optimal antibody pair (#20 and #46).

We next determined the optimal pair of mAbs for antigen-capture ELISA by evaluating all possible combinations of immobilized and labeled mAbs (**Figure 5A**). The combinations of #46/#5, #20/#13, #29/13, #46/#45, and #46/#20 exhibited higher S/N ratios than other pairs (**Figure 5B**). Based on these data, we constructed immunochromatographic strips using five pairs of mAbs (**Figure 6A**). We searched for the combinations yielding the highest color intensity at the positive control at the appropriate position with the lowest overall background on the rest of the membrane. We found that immobilization of mAb #46 and colloidal gold conjugation of #20 was the optimal combination for ICT (**Figures 6B,C**). Thus, we selected mAbs #46 and #20 for further investigation.

### No Evidence of Cross-Reactivity of Anti-MERS-CoV-NP Antibody to Other Human Coronaviruses

**FIGURE 7:**
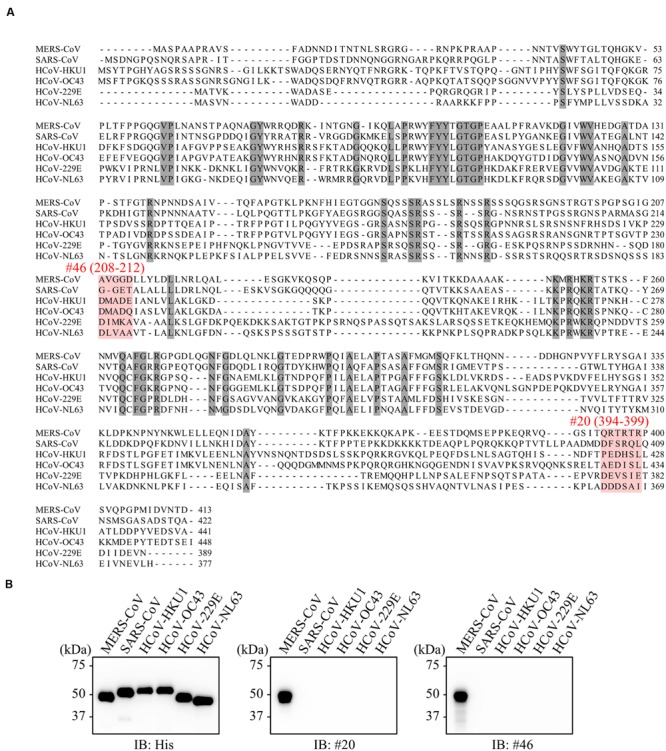
**No evidence of cross-reactivity with mAbs of other human coronaviruses. (A)** Multiple sequence alignments of human coronavirus N proteins. Shaded positions represent conserved residues among the sequences. Dashes indicate gaps in the aligned sequences. **(B)** Specificity of selected mAbs. His-tagged NPs derived from several human coronaviruses were produced in the wheat germ extract system. Reactivity of generated mAbs was validated by immunoblot analysis using either anti-His or the indicated antibodies.

We next investigated the specificity of our newly developed mAbs. Multiple alignment of NPs derived from various human coronaviruses revealed that the amino-acid sequences targeted by the selected antibodies were specific to MERS-CoV (**Figure 7A**). Consistent with these data, immunoblot analysis revealed that our mAbs did not recognize NPs from other coronaviruses (**Figure 7B**).

### Reactivity of Antibodies to Divergent Strains of MERS-CoV

**FIGURE 8:**
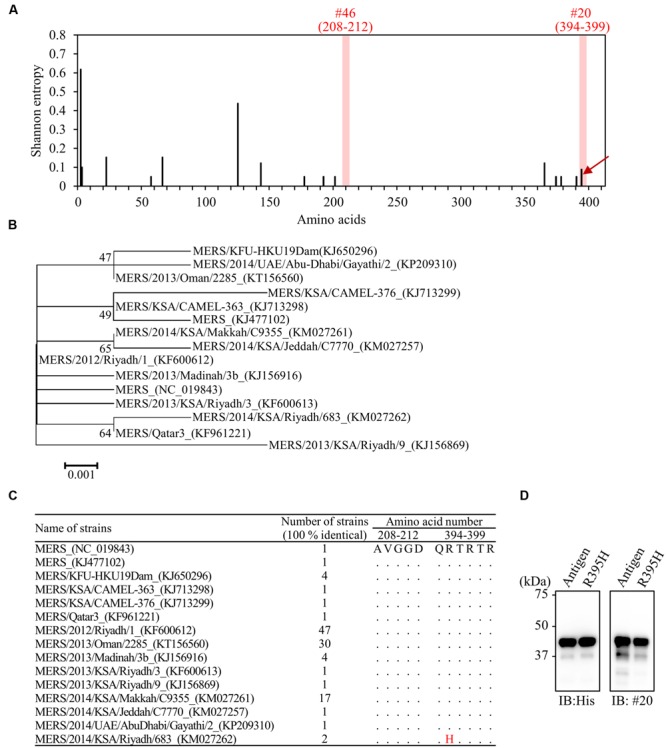
**Comprehensive detection of MERS-CoV isolates by the generated mAbs. (A)** Distribution of amino-acid variation of NP in 113 MERS-CoV strains. Shannon entropy, as a quantitative measure of variation, was calculated for each amino-acid residue of MERS-NP. Arrow indicates amino-acid mutation in the epitope of mAb (#20) from two MERS-CoV strains. **(B)** Phylogenetic analysis of MERS-CoV based on multiple sequence alignment of NP. The unrooted phylogeny is generated from amino-acid sequence alignments of nucleocapsid proteins based on the maximum-likelihood method. Sequences with identity of 100% are removed from the dataset. Bootstrap values (1,000 replicates) are indicated around the branches. The scale bar represents amino-acid substitutions per site. **(C)** Identification of amino-acid substitution in the antigenic epitope in 15 identical strains. Dots indicate sequence identity relative to the prototype strain. The R395H substitution was noted in two strains. **(D)** Reactivity of mAb (#20) to MERS-NP (122–413) derived from the prototype and a mutant strain harboring R395H. Reactivity was determined by immunoblot analysis.

To estimate the reactivity of mAbs with different strains of MERS-CoV, we compared the amino-acid sequences of epitopes between 113 isolated strains. Shannon entropy and phylogenetic analysis revealed that the amino-acid sequence of MERS-NP is highly conserved (**Figures 8A,B**). Notably, no obvious amino-acid mutation was observed in the binding region of #46 (**Figures 8A,C**). However, the C-terminal amino acid sequence targeted by #20 was rather variable, containing a specific mutation (R395H) in two strains (**Figures 8A,C**). Therefore, we performed site-directed mutagenesis to introduce the R395H mutation into MERS-NP and examined the effect on the reactivity of #20 antibody by immunoblot analysis. The results showed that mAb #20 could still detect the R395H mutant (**Figure 8D**).

### Reactivity of mAbs to Virion Nucleocapsid Protein

**FIGURE 9:**
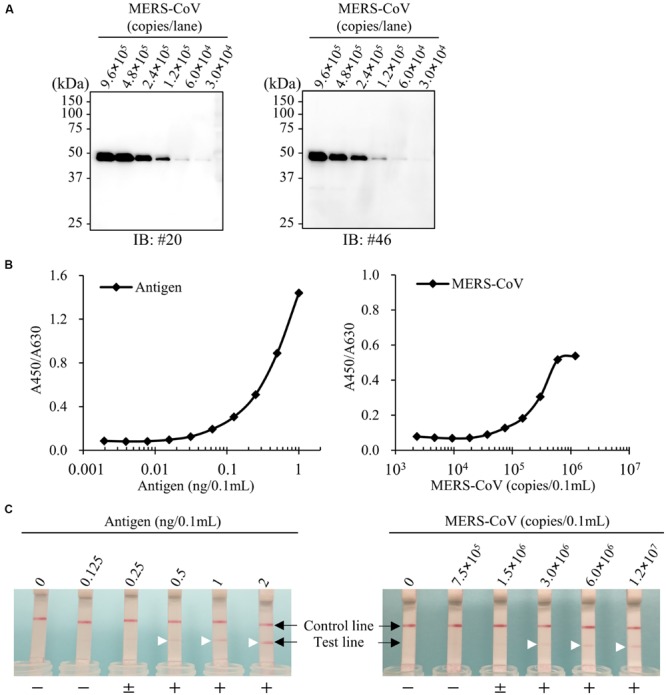
**Establishment of antigen detection assays for MERS-CoV. (A)** Immunoblot analysis for virus particles of MERS-CoV. Cell culture medium of MERS-CoV-infected Vero-TMPRSS2 cells were collected and analyzed by immunoblotting with the newly developed anti-MERS-NP mAbs. **(B,C)** Detection limit of ELISA and ICT. Recombinant antigen protein and MERS-CoV were subjected to either ELISA or ICT analysis. + and - indicate positive and negative detection, respectively; ± means moderately positive. Arrowheads show positive test line.

We next performed immunoblot analysis with virions released into the cell-culture supernatant of MERS-CoV infected cells. Our mAbs detected a 45 kDa protein band consistent with the molecular mass of the MERS-NP (**Figure 9A**). No other bands were detected by the mAbs, demonstrating their specificity for MERS-NP. Thus, our newly developed antibodies could detect NP antigen derived from MERS-CoV virions as well as recombinant NP.

### Sensitivity of Antigen-Capture ELISA

Using the optimal antibody pair (#46 and #20) identified above, we determined the detection threshold for antigen recognition by antigen-capture ELISA. Our results revealed that our system was highly sensitive to recombinant antigen, capable of detecting the protein at a concentration of 0.0625 ng/0.1 mL (**Figure 9B**). In parallel, we investigated the detection limit of ELISA for MERS-CoV virions permeabilized by addition of NP-40. The detection limit of the system was 1.5 × 10^5^ copies/0.1 mL (**Figure 9B**).

### Detection Limit of ICT for MERS-CoV

Finally, we evaluated the sensitivity of ICT for MERS-CoV. For this purpose, serial dilutions of purified NPs were subjected to ICT. The results revealed that ICT was highly sensitive, with a detection limit of 0.5 ng/0.1 mL. We next examined the detection limit for virions prepared in phosphate buffer containing 1% NP-40. ICT could detect virions at a concentration of 3.0 × 10^6^ copies/0.1 mL (**Figure 9C**).

## Discussion

We report the development and prospective evaluation of an ELISA and ICT for the quantitative and qualitative detection of MERS-CoV-NP antigen. Our newly developed assays provide rapid detection of a broad range of NP antigens derived from various isolates of MERS-CoV. Because NP is a principal structural protein that is more abundantly expressed than other MERS-CoV antigens, targeting NP for clinical diagnosis is both reasonable and practical (Chen et al., 2015).

The quality of a mAb is determined mostly by the antigen design, adjuvant selection, and antigen quality (Leenaars and Hendriksen, 2005; Stills, 2005). In particular, preparation of high-quality antigen is essential for creation of a specific mAb. There are several methods for preparing immunizing virus antigen, including synthetic peptide, virus-like particle (VLP), and purified recombinant protein. Synthetic peptides containing predicted immunogenic epitope(s) are the most widely used way to create immunogens derived from virus antigens. However, synthetic peptides are commonly linear and therefore often do not represent the native features of antigens that originate from the actual spatial structures of viral components.

On the other hand, cell-based protein expression systems such as *Escherichia coli* or baculovirus-insect cell systems are also widely used and popular approaches. Although a number of cellular proteins have been successfully generated using a cell-based approach, it is not feasible to use these strategies to generate viral proteins, because many viral antigens including human coronavirus N proteins are generally insoluble and aggregate in inclusion body fractions (Das and Suresh, 2006). Moreover, viral proteins are often cytotoxic, and expression of these proteins, particularly at high levels, can result in cell death (Cheng et al., 2006). By contrast, the cell-free protein production system permits synthesis of toxic proteins that otherwise cannot be produced in live cells. Wheat germ extract, a commonly used cell-free approach, utilizes a eukaryotic translation system to synthesize properly folded and biologically active proteins similar to those expressed in mammalian cells (Endo and Sawasaki, 2005, 2006; Goshima et al., 2008). These advantages highlight the suitability and availability of the wheat germ cell-free system for the generation of antigenic proteins that can be used to immunize animals and generate mAbs (Matsunaga et al., 2014).

The results of this study clearly demonstrate the advantages of using the wheat germ cell-free system for creating mAbs against MERS-CoV-NP. In general, mAbs can be divided into two groups, conformation-sensitive and -insensitive. Antibodies can be suitable for immunoprecipitation, immunoblotting, or both. A conformation-insensitive (immunoblotting-oriented) mAb can detect denatured linear antigen or peptide immobilized on membrane, whereas a conformation-sensitive (immunoprecipitation-oriented) mAb typically recognizes a native tertiary structure of antigen protein (Mancia et al., 2007; Takeda et al., 2015). In this study, we used the wheat germ system to synthesize MERS-NP antigen as a soluble protein, and consequently we were able to produce antibodies that specifically targeted antigenic epitopes located on the surface of MERS-NP. Accordingly, our newly developed antibodies were suitable for immunoprecipitation, indicating that they are sensitive to protein conformation. Because antibodies binding to protein surface epitopes are suitable for antigen detection assays such as ELISA and ICT, our newly create antibodies can be used in various immunological assays.

Other than MERS-CoV, many types of human coronaviruses are related to respiratory diseases. These include coronaviruses such as HCoV-229E, -OC43, -NL63, and -HKU1, which are responsible for common cold and upper respiratory diseases, as well as SARS-CoV, which causes life-threatening pneumonia. Therefore, it is important to create mAbs with high specificity for MERS-CoV in order to rule out other coronavirus infections. Previous reports indicated that the NTD of NP of many human coronaviruses share common sequences (Yu et al., 2005; Chang et al., 2014). Therefore, we used a recombinant MERS-CoV-NP devoid of conserved regions as an immunogen to produce mAbs. Consequently, our newly developed mAbs can recognize species-specific amino-acid sequences in MERS-CoV. Consistent with this, the mAbs did not cross-react with NPs derived from other human coronaviruses. Viral species can be determined by structural characterization of the capsid/nucleocapsid (Gelderblom, 1996). Therefore, we suspect that highly species-specific mAbs were obtained due to the appropriate design and native properties of the antigen.

Development of international trade and worldwide travel brings about significant risk of the spread of emerging infectious diseases, including MERS-CoV. Needless to say, there is no obvious boundary between virus-free countries and those facing endemics. Thus, the establishment of rapid and reliable laboratory diagnostic tests for these pathogens is an urgent matter in all countries. The most widely used current diagnostics for MERS-CoV involve detection of virus nucleic acids by qRT-PCR in the laboratory (Corman et al., 2012a,b). Although qRT-PCR is a sensitive and powerful tool for obtaining evidence of virus infection, it requires specialized lab equipment and expertise with molecular technology; moreover, due to the time required for the enzymatic reaction, it is rather time-consuming. These disadvantages prevent qRT-PCR from being used in POCT. Therefore, case-oriented comprehensive tests should be conducted using multiple diagnostic assays.

Enzyme-linked immunosorbent assay and ICT are two major clinical tests used to detect viral antigens. Both methods employ pairs of mAbs used as capture and detection antibodies. The optimal combination of capture and detection antibodies should be thoroughly investigated before the test kits are assembled. In this study, the utilization of natively folded antigen protein to evaluate each test allowed us to identify the optimal pair of antibodies for the detection of MERS-CoV-NP. Our antibody set is suitable for detecting MERS-CoV-NP by either ELISA or ICT.

At the early stage of illness, high titers of infectious virions and virus antigens are present in the lower respiratory tract (LRT) and sputum of patients. The viral loads in LRT and expectorated sputum of patients during acute infection are more than 10^6^ copies/mL (Drosten et al., 2013; Kapoor et al., 2013; Corman et al., 2015), making it feasible to detect viral antigens of MERS-CoV for clinical diagnosis at an early stage of infection. The current version of our assay can detect 0.0625 (ELISA) or 0.5 ng (ICT) of recombinant N protein and 1.5 × 10^5^ (ELISA) or 3.0 × 10^6^ viral copies (ICT), ensuring its feasibility in practical clinical tests.

Other groups have also developed antigen-capture ELISA and ICT for detection of MERS-NP antigen (Chen et al., 2015; Song et al., 2015). Song et al. used synthetic peptides as immunogens to raise an NP-specific mAb. The other group used recombinant N proteins as antigens, but did not perform precise epitope mapping (Chen et al., 2015). On the other hand, we developed structure-sensitive mAbs against MERS-NP and thoroughly investigated their targeting epitopes. Bioinformatic analysis based on a phylogenetic approach further revealed that our newly developed mAbs can detect NP antigens derived from existing isolates of MERS-CoV.

In summary, we developed a novel antigen-detection assay using newly created mAbs for the rapid and reliable assessment of NP antigen of MERS-CoV. Further evaluations using actual patient samples warrants the usability and benefit of this assay for the clinical diagnosis of MERS-CoV infection.

## Author Contributions

YY designed and performed the research, analyzed the data, and wrote the manuscript. SM and SF performed the research, contributed the virus preparation, analyzed the data. SM performed the research, analyzed the data. YM performed the bioinformatics analysis. HK, HK, MT, and TC edited the manuscript. AR directed the research, analyzed the data, and wrote the manuscript.

## Conflict of Interest Statement

The authors declare that the research was conducted in the absence of any commercial or financial relationships that could be construed as a potential conflict of interest.
